# Coronary Revascularization in Patients With Stable Coronary Artery Disease: The Role of Imaging

**DOI:** 10.3389/fcvm.2021.716832

**Published:** 2021-10-28

**Authors:** Danilo Neglia, Natallia Maroz-Vadalazhskaya, Nazario Carrabba, Riccardo Liga

**Affiliations:** ^1^Cardiovascular Department, Fondazione CNR Regione Toscana G. Monasterio, Pisa, Italy; ^2^Sant'Anna School of Advanced Studies, Pisa, Italy; ^3^Postgraduate Division, Belarusian State Medical University, Minsk, Belarus; ^4^Cardiothoracovascular Department, Careggi Hospital, Florence, Italy; ^5^Cardiothoracic and Vascular Department, University Hospital of Pisa, Pisa, Italy; ^6^Dipartimento di Patologia Chirurgica, Medica, Molecolare e dell'Area Critica, Università di Pisa, Pisa, Italy

**Keywords:** revascularization, coronary artery disease, cardiac imaging, prognosis, COVID-19

## Abstract

In the last decades, the effective management of some cardiovascular risk factors in the general population has led to a progressive decrease in the prevalence of coronary artery disease (CAD). Nevertheless, coronary heart disease remains the major cause of death in developed and developing countries and chronic coronary syndromes (CCS) are still a major target of utilization of non-invasive cardiac imaging and invasive procedures. Current guidelines recommend the use of non-invasive imaging in patients with CCS to identify subjects at higher risk to be referred for invasive coronary angiography and possible revascularization. These recommendations are challenged by two opposite lines of evidence. Recent trials have somewhat questioned the efficacy of coronary revascularization as compared with optimal medical therapy in CCS. As a consequence the role of imaging in these patients and in in patients with ischemic cardiomyopathy is under debate. On the other hand, real-life data indicate that a consistent proportion of patients undergo invasive procedure and are revascularized without any previous non-invasive imaging characterization. On top of this, the impact of COVID-19 pandemic on the sanitary systems caused a change in the current management of patients with CAD. In the present review we will discuss these conflicting data analyzing the evidence which has been recently accumulated as well as the gaps of knowledge which should still be filled.

## Introduction

In the last decades, the prevalence of obstructive coronary artery disease (CAD) and significant myocardial ischemia has been progressively decreasing in stable patients (1–4). As a result of the lower prevalence of disease, the accuracy of most of the currently available diagnostic strategies for the evaluation of patients with chronic coronary syndromes (CCS) has been steadily decreasing. Both non-invasive and invasive tests for the evaluation of (hemodynamically significant) CAD have recently shown a low diagnostic yield (1, 5, 6). The effective management of some cardiovascular risk factors, such as the promotion of healthy lifestyles and reduction of smoking, widespread use of anti-hypertensive and lipid-lowering treatments may have accounted for this trend. Nevertheless, new risk factors are emerging, such as diabetes, obesity, and metabolic syndrome (7, 8) which are, in turn, independently linked to CAD, as well as coronary heart disease (CHD) mortality and heart failure (HF). As a result, CHD remains the major cause of death in developed and developing countries (9). Accordingly, accurate strategies are now required to evaluate patients with suspected CAD or heart failure, and to characterize their clinical risk profile as well as the presence and extent of CAD. Efforts should be devoted in identifying those subjects at higher risk of cardiac events who might benefit more from optimal and targeted medical therapy alone or associated with coronary revascularization.

## Coronary Revascularization of Obstructive CAD: Is That Still the Major Goal?

In patients with CCS, consistent evidence has challenged the concept that revascularization might improve prognosis more than optimal medical treatment (OMT) (10, 11). The relative lower prevalence of severe disease in current populations as compared with the past (11, 12) and the prognostic efficacy of OMT targeted to some established risk determinants of coronary atherosclerosis and/or ischemia (13), could somehow explain these findings. Nevertheless, the established concept that revascularization could improve symptoms and prognosis in patients with obstructive CAD is still guiding clinical practice. Accordingly, referring patients with stable symptoms to invasive coronary angiography (ICA) based only on clinical suspicion and without an objective documentation of inducible myocardial ischemia is still a common choice (14).

Recent publications have shown that the pre-test probability (PTP) figures in the previous “European Society of Cardiology” (ESC) guidelines were grossly overestimated (15). The current ESC guidelines for the management of CCS (3) use updated prediction models, showing values of probability of significant CAD which are around 1/3 of those previously reported, and recommend the preliminary definition of the PTP of disease. In patients with PTP > 15% non-invasive cardiac imaging is strongly encouraged to detect either “high risk” myocardial ischemia or obstructive CAD before considering invasive procedures. Such recommendations are still based on classical evidence coming from historical observational studies suggesting that “significant ischemia” in the absence of extensive scar would benefit from revascularization (16). This concept has been challenged by recent randomized trials such as the “International Study of Comparative Health Effectiveness with Medical and Invasive Approaches” (ISCHEMIA) trial, in which patients with suspected CAD and moderate-to-severe ischemia were randomized to either an invasive strategy (ICA and revascularization when feasible) plus OMT or a conservative management with OMT alone (11). Over a 5-years follow-up there was no significant difference in event-free survival among the two strategies leading to reconsider the indication to invasive procedures in patients with suspected CAD and moderate-to-severe ischemia. These results were the last of a series of evidence coming from randomized trials progressively downplaying the role of functional testing to stratify the risk and guide the management of contemporary populations of patients with CCS and low prevalence of CAD and low overall risk of future cardiac events (17, 18). Despite these evidences (11, 17, 18) there are also reasons to question the simplistic conclusion that revascularization is useless in patients with documented extensive ischemia.

First, in most of these trials multiple, different and not necessarily equivalent modalities were used for the evaluation of myocardial ischemia (1, 6). Second, some relevant patients' categories (i.e., anatomical left main stenosis, proximal LAD stenosis, LV systolic dysfunction, and severe symptoms at baseline) have been excluded from major randomized controlled trials (RCT) on CCS, likely reducing the number of those “high risk” patients who could have benefited more from revascularization. Third, the existence and exact degree of the “specific” burden of ischemia above which revascularization would possibly improve prognosis over OMT is still a matter of intense speculation. According to current clinical guidelines, revascularization should be indicated in patients with an ischemic burden > 10% of the LV (3). In a recent large observational study, the association between early PCI or CABG (performed <90 days from the first evaluation), the ischemic burden and all-cause mortality has been investigated in a large population of patients who underwent single-photon emission computed tomography (SPECT) myocardial perfusion imaging (19). At survival analysis, when patients were stratified according to the extent of ischemia, coronary revascularization (either with PCI or CABG) was associated with decreased mortality in patients with ischemia involving >15% of the myocardium. These findings were consistent with prior appraisals (16) and fixed a higher threshold for ischemia severity which might be associated with revascularization benefit.

A final major limitation of these trials was that patient management was not guided by imaging results but left at the discretion of the referring physicians. Thus, the “appropriateness” of coronary revascularization, as defined by treating obstructive CAD associated with significant downstream ischemia and deferring revascularization in the other cases, could not be determined. In the ISCHEMIA study, ischemia was documented only by stress ECG in almost 25% of patients (11). Moreover, ICA was complemented by FFR measurements in only 20% of patients randomized to the invasive strategy (11), while invasive confirmation of the hemodynamic severity of a coronary stenosis by FFR is required by current Guidelines (3). Moreover, other intravascular imaging approaches are increasingly recognized as effective means to guide revascularization procedures (20). As a matter of fact, in a significant percentage of patients the assignment of ischemia to a specific coronary territory could not be possible, and the “appropriateness” of revascularization difficult to be established. In this respect, consistent evidence has been recently accumulating, demonstrating that while “appropriate” revascularization is prognostically beneficial, inappropriate procedures may predispose to adverse cardiac events (12, 20, 21).

A final consideration applies to the prognostic efficacy of OMT when targeted to the coronary atherosclerotic and ischemic processes. The diffusion of computed tomography coronary angiography (CTCA) as a first screening test in patients with CCS has increased the recognition of coronary atherosclerosis even in its earlier non-obstructive stages. Including CTCA in the diagnostic process of patients with CCS has been shown to improve outcome as compared to standard of care (18), independently from downstream referral to ICA or revascularization procedures. A possible reason behind these findings is that the evaluation of coronary anatomy with CTCA may allow a better risk-stratification of CCS patients than ischemia imaging, possibly unmasking high-risk patients' categories that would be missed by functional techniques. For instance, even in the absence of critical focal lesions, the presence of diffuse non-obstructive CAD may still cause symptoms and myocardial ischemia (22) and may be associated with adverse prognosis, deserving aggressive OMT to prevent future events (23).

**Figure 1:**
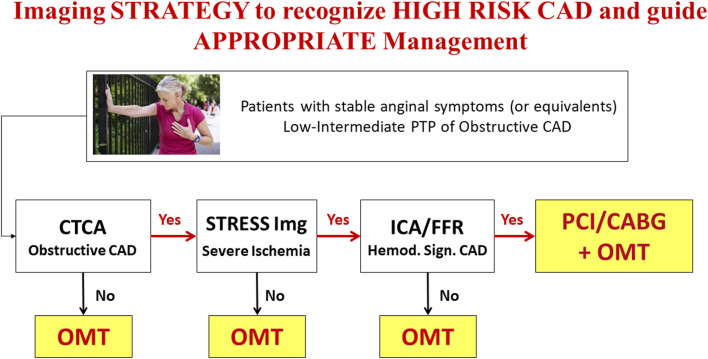
Anatomical and functional imaging integrated strategy to recognize “High Risk” CAD and guide management in patients with low-intermediate PTP of obstructive CAD (see text for details). OMT, Optimal Medical Treatment.

In conclusion, current evidence is in favor of an accurate assessment of coronary anatomic and ischemic burden in patients with CCS for risk-stratification and targeting of OMT (3). In patients with obstructive CAD, in whom diameter stenosis >90% or severe inducible myocardial ischemia is documented, coronary revascularization on top of OMT might be still the best option, to both control symptoms (24), and possibly improve outcome. The combined assessment of coronary anatomy and myocardial ischemia by an appropriate non-invasive imaging strategy (25–28) may represent the ideal tool for patients' characterization and a gatekeeper to inappropriate invasive procedures (Figure 1).

## Management of Patients WIH Stable CAD: Role of Imaging

The choice of the best diagnostic strategy to be followed in specific categories of patients with CCS is still a matter of debate, given the presence of a limited number of comparative prospective studies in patients with suspected or known CCS. Despite classically considered interchangeable for diagnostic purposes, anatomical and functional imaging techniques allow the assessment of distinct aspects of CAD that are associated with specific risks and may possibly require individualized treatments. Current guidelines in CCS (3) recommend a stepwise evaluation of patients largely based on non-invasive imaging modalities. While CTCA is the reference technique for the assessment of coronary atherosclerosis, stress imaging—either with MPI or wall motion imaging (WMI) —is employed to quantify the burden of inducible myocardial ischemia. Contemporary populations of patients with CCS referred for screening have indeed a lower prevalence of significant CAD than previously expected (1, 29), thus candidates to imaging screening should be carefully selected to avoid useless risks and costs. As underlined by current guidelines recommendations, the evaluation of PTP of disease should be integrated with other clinical parameters to better identify patients with intermediate or high likelihood of obstructive CAD who should be submitted to imaging tests (3, 30). Accordingly, only patients judged at high risk of future major cardiac events (>3% event-rate per year) after non-invasive imaging assessment should be referred to ICA (3). When a non-invasive imaging test provides an uncertain result a second test is recommended. This is particularly important when an anatomical imaging such as CTCA is performed first. The evidence of obstructive CAD (in the absence of left main or three vessel disease and/or proximal LAD obstruction) may be not sufficient to proceed to ICA. The demonstration of significant inducible myocardial ischemia by an additional stress imaging test will better identify those patients who will benefit more from invasive procedures (Figure 1), without forgetting the case of “balanced ischemia,” the Achilles heel of stress imaging in case of multivessel CAD (31).

The EVINCI-Outcome study, enrolling patients with stable angina submitted to both CTCA and stress imaging before ICA, provides evidence on the role of imaging to define appropriate treatment and the potential effects on prognosis in a contemporary population of patients with CCS and a low prevalence of disease (30%) (12). Patients with CAD in whom early revascularization was defined appropriate, because performed in the presence of significant inducible ischaemia and deferred in its absence, had an outcome not significantly different from that of patients without CAD. Conversely, patients with CAD who were revascularized despite no evidence of ischaemia or in whom intervention was deferred despite evidence of ischaemia had approximately a three-fold higher risk of major coronary adverse events than patients with no CAD. Thus, the study results suggested that in a population with low prevalence of significant CAD, a strategy using CTCA as the first test is reasonable. Nevertheless, when anatomical disease is found by CTCA, functional imaging before ICA is necessary to identify those patients with significant inducible ischaemia, who have most to gain by revascularization. In a health-economics analysis of the same study (32) it was shown that combined non-invasive strategies with CTCA and stress imaging are both cost effective as gatekeepers to ICA and to select candidates for early revascularization.

**Figure 2:**
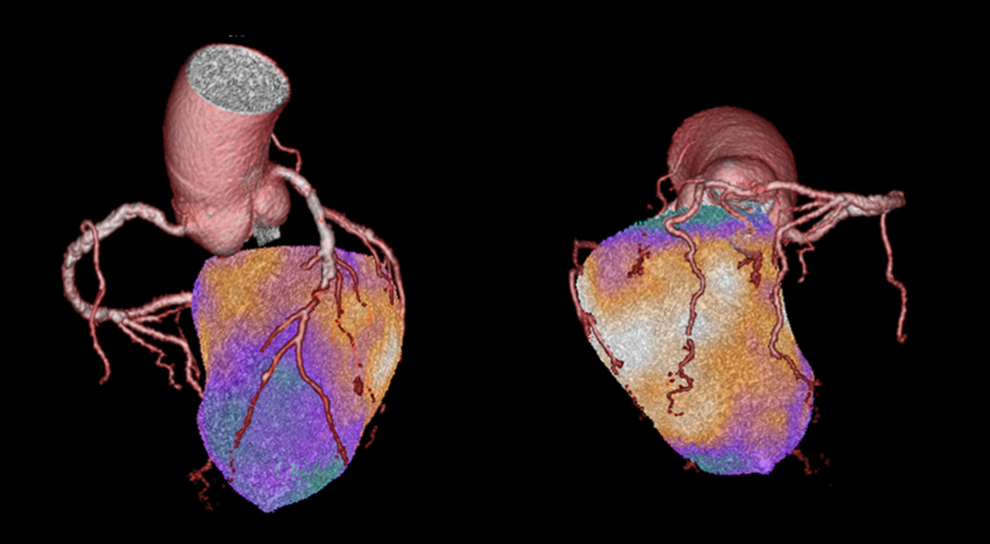
This is a case from the EVINCI Hybrid Imaging cohort (24). A 72-year-old gentleman with atypical chest pain and normal LV systolic function. His pre-test probability of obstructive CAD was 34%. PET MPI with H215O was performed at rest and after adenosine stress, showing the presence of an extensive, entirely reversible, stress-induced perfusion defect involving the LV septum, and the entire apical region. CTCA revealed the presence of multiple significant coronary lesions of the LAD with a sub-total mid occlusion, diffuse atherosclerosis of the LCx, and a significant (50–70%) stenosis of the mid RCA. On hybrid imaging, the entire perfusion defect was reassigned to the LAD, effectively changing the diagnosis from two-vessels to one-vessel disease. Imaging findings were later confirmed by ICA, showing two high-grade lesions in the LAD, and diffuse non-significant atherosclerosis in the other vessels.

Limited evidence exists on the possible additional role of combined anatomic/functional cardiac imaging in this setting. Nuclear stress imaging tests are well-suited to complement CTCA results also due to their recognized prognostic role (25, 33). Integration of functional information from MPI with anatomical description of coronary atherosclerotic disease is easily obtainable in 3D reconstructions by hybrid SPECT/CT and PET/CT imaging (34). The combination with CTCA performed with new generation scanners and specific acquisition protocols allows obtaining this combined information with an overall radiation dose between 4 and 10 mSv, making combined anatomic/functional cardiac imaging an appealing alternative to the classical single-imaging approach. Hybrid imaging, by directly assessing the functional significance of a coronary stenosis holds much promise for future clinical application in better selecting patients for invasive procedures. The clinical value of this approach has been recently explored in the population of the EVINCI trial (25). In this multicenter population of 252 patients with intermediate PTP of CAD, hybrid images have been obtained by 3D fusion of either SPECT/CTCA or PET/CTCA datasets and evaluated by independent observers. The presence of anatomical-functional “match” (inducible perfusion defect downstream an obstructive coronary lesion at CTCA) allowed recognizing significant CAD in 24% of patients, while a negative “match” excluded significant CAD in 41% of patients with an optimal diagnostic accuracy as compared with ICA (PPV 87% and NPV 88%). Moreover, because of the 3D evaluation of coronary anatomy and myocardial perfusion, hybrid imaging also allowed to reallocate perfusion defects to the appropriate coronary territory in 42% of patients and predicted subsequent revascularizations (Figure 2). These concepts have been further strengthened by recent evidence, indicating the incremental prognostic value of hybrid imaging over CTCA alone (35, 36).

An alternative to ischemia testing is the evaluation of the hemodynamic significance of the coronary stenosis at the time of CTCA by means of FFR_CT_ (26–28). The development of a CTCA-based index represented a relevant step forward to allow a complete anatomical-functional characterization of CAD by a single investigation, possibly reducing the downstream inappropriate referral to invasive coronary angiography (37). In fact, in the last years several studies have reported how the progressive refinements of computational fluid dynamics (CFD) models have brought to a radical increase of FFR_CT_ ability in unmasking the presence of invasively assessed hemodynamically significant coronary lesions (26, 27). FFR_CT_ seems particularly suited for the investigation of patients with suspected or known CCS, since it provides high accuracy for detection of hemodynamically significant CAD without additional radiation exposure and an improved cost-effectiveness if compared to the standard of care. However, some technical limitations of FFR_CT_ algorithms should be also acknowledged, including the remote and lengthy core-laboratory analysis currently required for the most tested and validated software. In this context, newest indexes have been proposed to overcome some of the limitations of traditional FFR_CT_ algorithms. Among those, virtual functional assessment index (vFAI), derived from the application of computational flow dynamics to standard CCTA datasets, had the main advantage to require a shorter computational time (20–30 min on average) to obtain the results in each case (28, 38). FFR_CT_ has been shown to mimic the results of invasive FFR and to be associated with inducible ischemia detected by stress SPECT (39, 40). On the other hand, vFAI has been validated both vs. invasive FFR (38) and against PET-derived absolute MBF measurements as integrated measures of global coronary fluid dynamics (28). However, additional research is needed to demonstrate their possible additional role in the diagnostic assessment of patients with CAD. Despite some intrinsic methodological limitations, FFR_CT_ and vFAI might be an appropriate tool for interrogating the functional significance of a coronary stenosis at CCTA and as a gatekeeper to invasive coronary angiography for revascularization.

Unfortunately, ICA is still commonly used for the diagnosis and to guide treatment without an adequate pre-selection of patients, resulting in high costs, frequent negative invasive studies or revascularization procedures mainly guided by anatomical findings. Moreover, even when stress imaging is performed its results are not fully considered in further management of the patients (5, 25, 41). Taken together the most recent findings underline the need to perform further trials which could assess the prognostic and cost-effectiveness impact of a management algorithm guided by appropriate combination of anatomical and functional imaging as compared with standard work-up.

Similarly, more research is needed to define whether the role of non-invasive imaging to guide management of patients with stable CAD could be reinforced by the capacity to characterize “high risk” coronary plaque features (42) which carry a relevant prognostic value, independently from the degree of obstruction and the associated ischemia (43, 44). Whether the recognition of “vulnerable plaques,” promoting a more aggressive medical treatment and/or guiding targeted interventional procedures, could improve outcome is an open research question (45). Moreover, in the near future, the optimal risk stratification and management in the single individual could be improved by a machine learning (ML) approach. A relevant number of imaging variables can be derived automatically and merged with clinical variables obtained by digitalized health records to feed artificial intelligence based decision support systems able to stratify prognosis and guide effective treatment (46, 47).

## Management of Patients With Ischemic Heart Failure: Role of Imaging

Ischemic cardiomyopathy (ICM) is generally identified by the presence of significant left ventricular (LV) systolic dysfunction, as defined by LV ejection fraction (EF) lower than 40%, associated with the presence of extensive CAD (48). While such patients have been almost systematically excluded from the most recent randomized controlled trials on the management of subjects with CCS, complete coronary revascularization is still a class I indication in this population according to current ESC guideline recommendations (49). Most of the evidence favoring revascularisation in patients with ICM derives from old observational studies (50), while weaker evidence comes from RCTs. In particular, the assessment of myocardial viability by non-invasive imaging is still advocated in these patients to decide on the need of coronary revascularization (3, 49, 51), based on the assumption that in the presence of relevant burden of hibernating myocardium coronary revascularization would result in LV function recovery and lead to a significant prognostic benefit (50). However, also in patients with ICM recent RCT provided conflicting results (52–54).

The Surgical Treatment for Ischaemic Heart Failure (STICH) trial is so far the largest RCT that has evaluated the impact of surgical coronary revascularization in patients with ICM. Of the 1,212 patients enrolled, 610 underwent coronary artery bypass graft (CABG) on top of OMT, while 602 patients were randomized to OMT alone (55). Despite the overall results of the trial were in favor of CABG (death rate 40.5% with CABG vs. 49.3% with OMT, *P* = 0.006), this difference was obtained only after extensive follow-up, because the early increase in mortality related to cardiac surgery was offset by beneficial effects only after >4 years. The pre-specified viability sub-study of STICH included the 601 patients with available information on the presence and extent of myocardial viability, as obtained through single-photon emission computed tomography (SPECT) or stress echocardiography (53, 56). While patients revascularized despite the absence of significant LV viability had the worst prognosis (overall survival 49 vs. 63%), this difference disappeared after correcting for baseline clinical variables (53). However, several major limitations of the study do not allow any conclusive statement on the topic. First, the imaging protocols employed for the assessment of myocardial viability were highly inhomogeneous with, for instance, five different SPECT protocols allowed. Moreover, since patients were not randomized according to the results of viability imaging only indirect evidence on the role of viability imaging in ICM can be inferred. In addition, it is tempting to speculate that the use of more accurate non-invasive imaging modalities, such as cardiac magnetic resonance imaging and positron emission tomography (PET), would have allowed a better characterization and risk-stratification of patients, likely translating into improved patients' management and possibly outcome.

The Positron Emission Tomography and Recovery Following Revascularisation (PARR-2) study randomized patients with ischemic heart failure to either a viability-guided management or standard care (52). Myocardial viability was evaluated through fluorodeoxyglucose (FDG) PET imaging and the likelihood of LV function recovery after revascularization was estimated based on the burden of hibernating myocardium. The overall results of the study were neutral, with a similar event-rate in patients randomized to PET imaging as compared to standard care (30 vs. 36%, *P* = 0.16). However, at further analysis, patients with a more significant burden of hibernating myocardium (>7% of the LV) showed a prognostic benefit from coronary revascularization (event-rate 13 vs. 56% in non-revascularized patients) (57). Moreover, when restricting the analysis to patients whose final management was adherent to PET results—revascularized only in the presence of hibernating myocardium and managed conservatively otherwise—a significant prognostic advantage of PET-based treatment was also observed (58).

Available evidence does not allow to make a conclusive statement of the role of viability-guided management in patients with ICM, posing the need of dedicated RCTs on the topic.

## Coronary Revascularization and Non-invasive Imaging in the COVID-19 Pandemics

The management of patients with new onset or worsening symptoms suggestive of CAD has become a challenge for medical personnel and healthcare systems with the worldwide spread of the COVID-19 disease, starting from the end of 2019. As a rule, the normal function of the cardiac catheterization laboratories has been altered to minimize the risk of personnel infection, to preserve hospital beds and to prioritize procedures known to have a higher impact on patients' outcome (59). A national survey among interventional cardiologists in the United States during the COVID-19 pandemic in 2020, identified unprecedented large-scale procedural deferrals, substantially reducing overall activity volumes in the cath-labs (60).

According to available figures, the monthly PCI volumes from March 15 to April 15, 2020 as compared with the same period of 2019 was reduced by 55% and this reduction was mainly due to deferral of PCI for stable CAD. These data have been most probably also influenced by evidence accumulated from the recent RCTs on the management of patients with CCS, downplaying the role of invasive management. Interestingly, interventional cardiologists perceived that non-invasive imaging studies were 14–24% more likely to be used to risk stratify patients instead of angiography.

**Figure 3:**
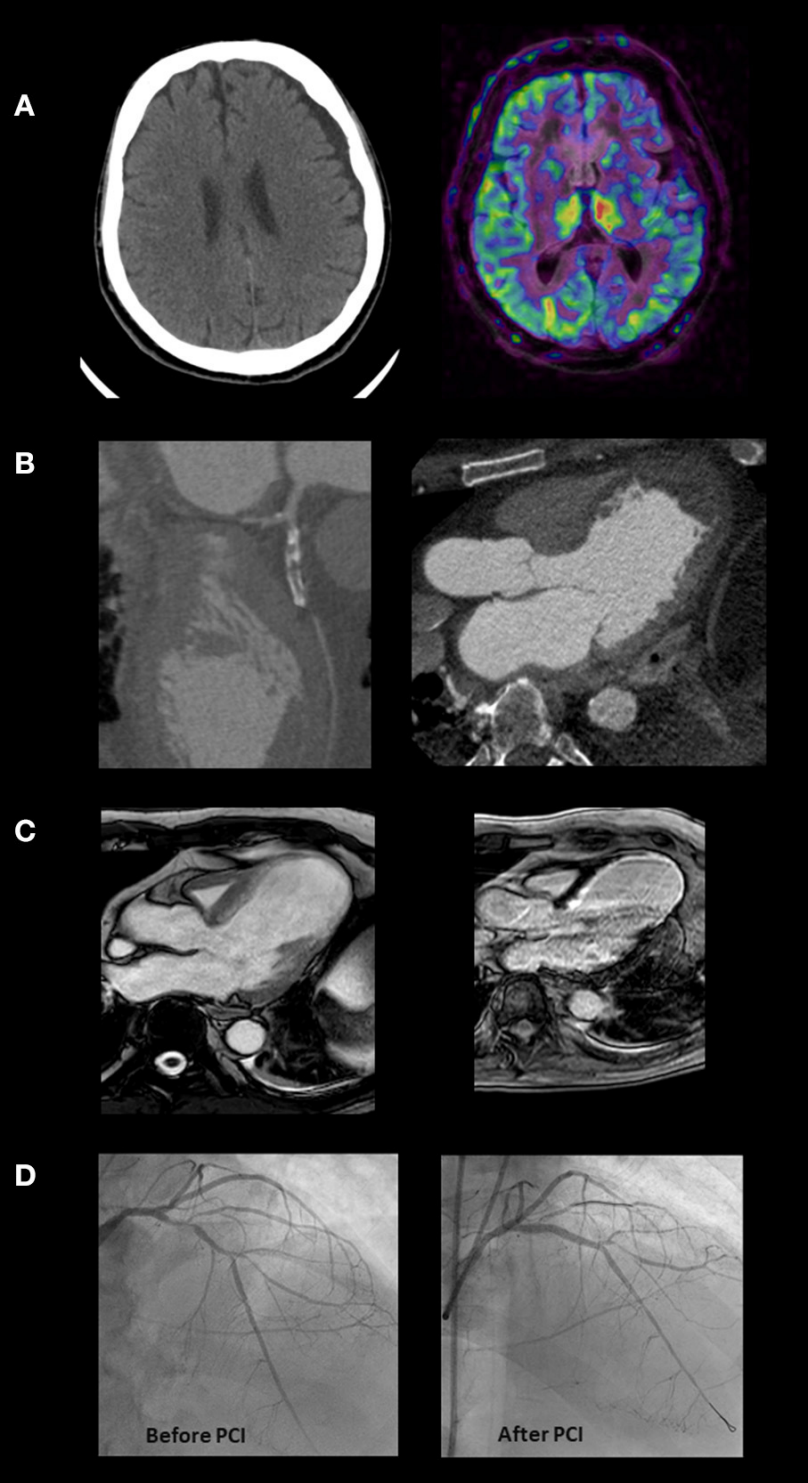
This is the case of a 64-year-old man admitted to the emergency department with symptoms of nausea and vomiting. He reported onset of chest pain and dyspnea 10 days before, during the COVID-19 lock-down. The ECG showed signs of an evolving anterior STEMI. The skull CT **(A)** evidenced limited left ischemic stroke and large left hygromatous flap. The CCTA **(B)** demonstrates an occluded proximal left anterior descending (LAD) coronary artery, a large apical aneurysm of the left ventricle and an apical thrombus. Considering the bleeding risk associated with dual antiplatelet therapy and heparin and the need to better define residual viability, invasive coronary angiography (ICA) was deferred starting oral anticoagulation. Apical thrombus was no more evident at cardiac MRI, performed 3 weeks later **(C)**. The exam showed a severe left ventricular dysfunction (LVEF 28%), and an extensive but not transmural scar in the territory supplied by the LAD. Based on this evidence, after 3 weeks more, the patient underwent ICA confirming LAD disease which was treated by PCI and drug eluting stenting **(D)**. Before hospital discharge the implantation of an ICD was planned but unfortunately a sudden cardiac death occurred soon after.

Interventional procedures with immediate mortality benefits, such as primary PCI in STEMI patients, remained prioritized in the hospital protocols (59). Nevertheless, preliminary analysis during the early phase of the COVID pandemic in March 2020 showed an estimated 38% reduction in U.S. cardiac catheterization laboratory STEMI activations (61), similar to a 40% reduction noticed in Spain (62), and the almost 50% reduction observed in Italy, which was paralleled by a substantial increase of the STEMI case fatality rate [risk ratio (RR) = 3.3, 95% CI 1.7–6.6; *P* < 0.001] compared to 2019 (63). While an increase of invasive procedures could have been expected, due to heightened environmental and psychosocial stressors or mimickers such as COVID-19 myopericarditis, the effects of the tendency to avoid medical care due to social distancing or concerns of contracting COVID-19 in the hospital could have prevailed. In an international survey promoted by the ESC in April 2020 on the impact of the COVID-19 pandemic on hospital admissions for cardiovascular emergency, such as STEMI, 80% of cardiologists (mainly interventional) and cardiovascular nurses felt there had been a decrease in STEMI presentations of at least a 40% reduction (64) (see the case presented in Figure 3).

The reduction in coronary revascularizations was mirrored by the drop in the use of non-invasive diagnostic procedures in patients with suspected CAD during the COVID-19 pandemics. In a worldwide survey assessing alterations in cardiovascular diagnostic procedure volumes and safety practices resulting from COVID-19, non-invasive and invasive cardiac testing volumes were obtained from participating sites for March and April 2020 and compared with those from March 2019 (65). Overall diagnostic procedure volumes decreased 64% from March 2019 to April 2020. Stress tests in patients with suspected CAD decreased by 78%, with slightly higher decrease for stress electrocardiography (84%) and echocardiography (83%) than for nuclear (73%) or CMR (72%) stress tests. Coronary angiography (invasive or computed tomography) decreased by 55%. Significantly greater reduction in procedures occurred for centers in countries with lower gross domestic product. Location in a low-income and lower–middle-income country was associated with an additional 22% reduction in cardiac procedures and less availability of personal protective equipment and telehealth.

International scientific societies, such as the European Association of Cardiovascular imaging of the ESC, issued recommendation on cardiac imaging during the COVID-19 pandemic with special attention on indications and prioritization (66). The general indications were that cardiac imaging should have been performed if appropriate and only if it is likely to substantially change patient management or be lifesaving and the imaging modality should be selected taking into consideration safety of both the patients and sanitary staff (67). Moreover, elective or follow-up exams might have been postponed. In this context, CTCA may offer specific advantages in patients with COVID-19 infection and elevated troponins for excluding or confirming CAD, substituting ICA (which has a higher associated exposure of all the members of the cardiac catheterization laboratory team). Moreover, CTCA can be considered in the COVID-19 pandemic in patients with chronic coronary syndromes and severe symptoms. Indications for stress echocardiography, as well as for other stress imaging techniques, are considered very limited in the COVID-19 pandemic and are recommended to be avoided in patients with acute infection. In these patients again coronary CTCA should be the preferred.

Dedicated studies will be warranted to assess whether some of the relevant changes in care delivery related with COVID-19 will last even after the pandemic period and whether these changes had an impact on cardiovascular outcomes.

## Conclusions

In the last decades, the role of coronary revascularization in patients with CCS has been progressively challenged. In this setting, non-invasive cardiac imaging, aiming at evaluating the presence of obstructive CAD and/or the extent of myocardial ischemic burden, is still recommended to identify individuals who might benefit more from an invasive management. Recent data have underlined the role of a sequential anatomical and functional diagnostic strategy to guide selective coronary revascularization and improve clinical outcome. This approach might be particularly suited in patients with ICM, in whom the risks of revascularization procedures are increased, and more targeted interventions are needed. Real-life data indicate, on the other hand, that a consistent proportion of patients undergo ICA and ultimately coronary revascularization without any previous characterization and proof of inducible myocardial ischemia, leading to inappropriate resources utilization and possibly a higher complications rate. During the COVID-19 pandemic the sanitary systems have been forced to reduce non-urgent interventional procedures including coronary revascularizations. While some useless procedures could have been avoided, there is clear evidence that unwanted delays have been accumulated in treating patients at high risk. This experience will probably further help to reconsider the effectiveness of current management of patients with CAD and prompt new trials to define the role of imaging guided revascularization vs. OMT.

## Author Contributions

DN, NM-V, NC, and RL contributed to conception of the review. DN wrote the first draft of the manuscript. NM-V, NC, and RL wrote sections of the manuscript. RL assembled the final version. All authors contributed to manuscript revision, read, and approved the submitted version.

## Conflict of Interest

The authors declare that the research was conducted in the absence of any commercial or financial relationships that could be construed as a potential conflict of interest.

## Publisher's Note

All claims expressed in this article are solely those of the authors and do not necessarily represent those of their affiliated organizations, or those of the publisher, the editors and the reviewers. Any product that may be evaluated in this article, or claim that may be made by its manufacturer, is not guaranteed or endorsed by the publisher.
